# Associations between tobacco and cannabis use and anxiety and depression among adults in the United States: Findings from the COVID-19 citizen science study

**DOI:** 10.1371/journal.pone.0289058

**Published:** 2023-09-13

**Authors:** Nhung Nguyen, Noah D. Peyser, Jeffrey E. Olgin, Mark J. Pletcher, Alexis L. Beatty, Madelaine F. Modrow, Thomas W. Carton, Rasha Khatib, Djeneba Audrey Djibo, Pamela M. Ling, Gregory M. Marcus

**Affiliations:** 1 Department of Medicine, Center for Tobacco Control Research and Education and Division of General Internal Medicine, University of California, San Francisco, San Francisco, California, United States of America; 2 Department of Medicine, Division of Cardiology, University of California, San Francisco, San Francisco, California, United States of America; 3 Department of Epidemiology and Biostatistics, University of California, San Francisco, San Francisco, California, United States of America; 4 Louisiana Public Health Institute, New Orleans, Louisiana, United States of America; 5 Advocate Aurora Health, Downers Grove, Illinois, United States of America; 6 CVS Health, Northbrook, Illinois, United States of America; University of Sao Paulo, School of Medicine, BRAZIL

## Abstract

**Background:**

Little is known about whether people who use both tobacco and cannabis (co-use) are more or less likely to have mental health disorders than single substance users or non-users. We aimed to examine associations between use of tobacco and/or cannabis with anxiety and depression.

**Methods:**

We analyzed data from the COVID-19 Citizen Science Study, a digital cohort study, collected via online surveys during 2020–2022 from a convenience sample of 53,843 US adults (≥ 18 years old) nationwide. Past 30-day use of tobacco and cannabis was self-reported at baseline and categorized into four exclusive patterns: tobacco-only use, cannabis-only use, co-use of both substances, and non-use. Anxiety and depression were repeatedly measured in monthly surveys. To account for multiple assessments of mental health outcomes within a participant, we used Generalized Estimating Equations to examine associations between the patterns of tobacco and cannabis use with each outcome.

**Results:**

In the total sample (mean age 51.0 years old, 67.9% female), 4.9% reported tobacco-only use, 6.9% cannabis-only use, 1.6% co-use, and 86.6% non-use. Proportions of reporting anxiety and depression were highest for the co-use group (26.5% and 28.3%, respectively) and lowest for the non-use group (10.6% and 11.2%, respectively). Compared to non-use, the adjusted odds of mental health disorders were highest for co-use (*Anxiety*: *OR = 1*.*89*, *95%CI = 1*.*64–2*.*18; Depression*: *OR = 1*.*77*, *95%CI = 1*.*46–2*.*16*), followed by cannabis-only use, and tobacco-only use. Compared to tobacco-only use, co-use *(OR = 1*.*35*, *95%CI = 1*.*08–1*.*69)* and cannabis-only use *(OR = 1*.*17*, *95%CI = 1*.*00–1*.*37*) were associated with higher adjusted odds for anxiety, but not for depression. Daily use (vs. non-daily use) of cigarettes, e-cigarettes, and cannabis were associated with higher adjusted odds for anxiety and depression.

**Conclusions:**

Use of tobacco and/or cannabis, particularly co-use of both substances, were associated with poor mental health. Integrating mental health support with tobacco and cannabis cessation may address this co-morbidity.

## Introduction

Tobacco and cannabis are among the most commonly used substances worldwide. In the U.S., 22.1% of adults reported using tobacco and 12.4% using cannabis in the past month in 2020 [1]. Co-use of tobacco and cannabis (defined in this study as concurrently using both substances in the past month) is also prevalent with 33% of people who use tobacco also reporting using cannabis in the past month [2,3]. Amid the expanding legalization of cannabis nationwide, use of cannabis and co-use of tobacco and cannabis have been increasing recently [3,4]. Studies showed that cannabis use increased over time and is 2–10 times more common among those with, versus without, cigarette smoking [3]. These trends raise concerns about unknown harms related to cannabis use in general and co-use of tobacco and cannabis in particular [5]. Co-use may increase exposure to toxicants and pose additive health risks [6,7]. It is also associated with increased risk for nicotine and cannabis dependence and poorer cessation outcomes for both tobacco and cannabis [8–10]. However, understanding of the association between co-use and health remains limited [5,11].

Use of either tobacco or cannabis are related to poor mental health [12–14] with the mixed literature on the direction of these relationships [14,15]. The extant work found that regular nicotine use is associated with increased risk of the onset and persistence of depression and anxiety [16–18]. Conversely, people with anxiety and depression are more likely to use cigarettes and other tobacco products (e.g., e-cigarettes, hookah) than those without these mental health disorders [19–21]. Current evidence regarding the association between cannabis and mental health is similar [13,14]. Cannabis use is two to three times higher among individuals with anxiety or depression compared with those without these conditions [22–24]. Also, cannabis use is a risk factor for the development of poor mental health and regular use may worsen depressive symptoms [25,26]. Previous studies have shown that mental health disorders are associated with heavy use and dependence of nicotine and cannabis as well as a decreased likelihood of successful cessation [27,28]. Thus, measuring mental health among people who do and do not use tobacco and/or cannabis will provide treatment implications for both mental health disorders and substance use cessation.

To date, previous research has predominantly examined the association of mental health with use of either tobacco or cannabis. Lacking are studies examining the association between co-use of both substances and mental health. The important gap to address is that whether people who use both substances are more or less likely to have mental health disorders than those who use single substances or neither of these substances. A study in the UK showed that people who used both tobacco and cannabis reported the highest prevalence of mental health treatment compared to those using single substances or not using these substances [29]. Likewise, the 2020 International Tobacco Control Survey found that co-use of tobacco and cannabis is associated with higher odds of having depressive symptoms compared to use of cigarette only among adults who smoked cigarettes in four countries (US, Canada, Australia, England) [30]. Research on co-use of tobacco and cannabis and mental health in US adult samples remains scarce. A study from a representative sample of US adults found that people with co-occurring cannabis use disorders and nicotine dependence were more likely to report psychiatric diagnoses and psychosocial problems than those with cannabis use disorders only or nicotine dependence only [31]. This study, however, collected data in 2004–2005. Given the changing product and policy landscape related to tobacco and cannabis in the US (e.g., emerging vaporized products, increasing cannabis legalization), more recent data on co-use and mental health are warranted. In addition, there has been a concerning rise in substance use and mental health problems related to social isolation and stressors during the COVID-19 pandemic. However, emerging evidence indicated mixed impacts of the pandemic with documenting both increases and decreases in substance use and mental health issues across samples. To inform efforts aimed at promoting public health well-being, it is more important than ever to address the association between use of tobacco and cannabis with mental health.

To address this gap, we analyzed a large dataset from an online cohort of US adults collected during 2020–2022, the COVID-19 Citizen Science Study (CSS) [32], leveraging recent substance exposures and previously validated mental health instruments. We aimed to examine the association between four exclusive patterns of tobacco and cannabis use (i.e., tobacco-only use, cannabis-only use, co-use of both tobacco and cannabis, and non-use) and mental health disorders (i.e., anxiety and depression). We hypothesized that co-use would be associated with higher odds of mental health disorders than use of tobacco or cannabis only and non-use.

## Materials and methods

### Study design and participants

This study analyzed data from the CCS study, a longitudinal cohort study hosted on the Eureka research digital platform (https://covid19.eurekaplatform.org/) [32]. The CCS study was launched in March 2020 and aimed to generate knowledge about long-term consequences of the COVID-19 pandemic based on participant-reported symptoms, behaviors, and disease occurrence. Study enrollment and follow-up are ongoing, without prespecified enrollment targets or end dates for enrollment or completion. Participation is remote, without geographic restriction. However, most participants are in the US and the participation from other countries is very low (<1%). Thus, the current study focused on only participants in the US.

This entirely digital study is hosted on Eureka, a digital research platform that produces mobile applications for clinical research built by investigators and developers at the University of California, San Francisco (UCSF). The Eureka platform is available to anyone who wants to conduct medical or health-related research, including university or other non-profit investigators, industry investigators or others. The app’s elements (e.g., electronic consent, surveys, and feedback) can be customized for individual studies. In January 2021, a web version of the CCS study was launched to enable participation options for people without a mobile phone or with concerns about downloading an app.

Using a convenience sampling method, participants were recruited through multiple channels, including email invitations to participants in other studies using the Eureka platform, press releases, word-of- mouth, social media sharing, and recruitment through partner organizations nationwide. Participants must be 18 years or older, agree to participate in English, provide consent, and register for a Eureka account. Participants were asked to complete baseline, daily, weekly, and monthly surveys about demographics, medical conditions, medications, and behaviors through the study app or website. Use of tobacco and cannabis was measured only once at baseline, while mental health was measured repeatedly in monthly surveys. There were no monetary incentives for participation in the study. Retention strategies include daily notifications, data visualizations, and intermittent study update blog posts. More details about the study procedures have been described elsewhere [32].

For this analysis, we included data collected between March 2020 (when the CCS study started) and April 2022 (the latest date when the analysis was conducted). Of 87,984 US participants who completed the baseline survey and provided data on tobacco and cannabis use, 53,843 participants (response rate of 61.2%) who completed at least one monthly survey on mental health were included in this analysis. The study duration for the participants ranged from 1–25 months, with an average length of follow-up of 8.5 ± 6.6 months. Results are reported in accordance with Strengthening the Reporting of Observational Studies in Epidemiology (STROBE) reporting guidelines.

### Ethical considerations

All participants provided informed written consent via a dedicated research mobile application to participate in the study. The CCS study was approved by the UCSF Institutional Review Board (IRB #17–21879).

### Measures

#### Mental health outcomes (Anxiety and Depression)

Participants were asked about their mental health repeatedly in monthly surveys, and thus, there were multiple assessments of mental health outcomes nested within a participant. While anxiety and depression symptoms frequently co-occur, they have differing but independent effects on functional impairment and disability [33]. Thus, we measured anxiety and depression separately using validated instruments.

Anxiety was measured by the 7-item Generalized Anxiety Disorder scale (GAD-7), a brief and validated screening measure for anxiety symptoms used in both clinical and community samples [33]. The GAD-7 captures the frequency (from 0-“Not at all” to 3-“Nearly every day”) of the seven symptom criteria for GAD over the past two weeks. Numeric responses to these questions were summed (range of 0–21). A cutoff of 10 or greater was used to dichotomize participants into having generalized anxiety disorder per recommended scoring guidelines [33].

Depression was measured by the 8-item Patient Health Questionnaire (PHQ-8), a brief and validated screening tool for a major depression episode within the past two weeks [34]. Responses for each item range from 0-“Not at all” to 3-“Nearly every day” with a total score with a range of 0–24. A score of 10 or greater was considered as having depression per recommended scoring guidelines [34].

#### Independent variable (Patterns of tobacco and cannabis use)

Use of tobacco and use of cannabis were reported separately in the baseline survey. Participants reported number of days they had used each tobacco product (i.e., cigarettes, e-cigarettes, and other combustible tobacco products like cigars/cigarillos) and number of days they had used any combustible or vaporized cannabis products during the past 30 days. Those reporting 0 days were classified as non-use of tobacco or cannabis. Those reporting 30 days were classified as daily use of a product. Based on their responses, participants were categorized into four groups: tobacco-only use, cannabis-only use, co-use (using both substances in the past 30 days), and non-use of either substance. These groups were created to investigate any potential independent effects of single substance use and joint effects of co-use on mental health disorders.

#### Covariates

Demographic characteristics and other covariates were collected at baseline. Age at registration was self-reported. Sex assigned at birth was self-reported as male and female. Gender identity was dichotomized into “cisgender” (i.e., man, woman) and “transgender and non-binary gender” (i.e., transgender, genderqueer, and other). Race and ethnicity were measured by combining two items: race (White, Black, Asian, Hawaiian/Pacific Islander, American Indian/Alaskan Native, or More than one race) and ethnicity (Hispanic or not). Responses were categorized into Hispanic, non-Hispanic White, non-Hispanic Asian, non-Hispanic Black, and non-Hispanic Other. Participants reported educational attainment in categories; the variable was recoded into “High school or less,” “Some college or college graduate,” “Postgraduate,” and “Other/Unknown.” Subjective social status was measured by the MacArthur Scale using a 1–10 stepladder with higher score indicating one’s higher perceived status in the social hierarchy [35]. Participants were also asked, “*On average*, *how often have you exercised (enough to breathe heavily and/or sweat) over the past year*?” with answer options ranging from “*Never of rarely” to “4 or more times a week*.” Participants also reported number of alcohol drinks they consumed in the past week. Physical activity and alcohol use were coded as continuous variables, corresponding to greater frequency of physical activity and alcohol consumption.

### Statistical analysis

Data were analyzed using STATA 15.0 (StataCorp LLC, College Station, TX, US). Participant characteristics were summarized for the total sample and for each pattern of tobacco and cannabis use. ANOVA tests were used to compare means for continuous variables across patterns of tobacco and cannabis use, nonparametric median tests were used to compare medians for non-normally distributed variables, and chi-square tests were used to compare proportions for categorical variables.

To examine the associations between the patterns of tobacco and cannabis use (a categorical independent variable) and mental health outcomes (the binary outcomes), we fit Generalized Estimating Equations (GEE) models to account for the nesting of multiple monthly assessments of anxiety and depression within each participant [36]. Participants with any number of either mental health assessment (as long as at least one was completed) were included in these analyses, with incorporation of every such assessment completed for each individual. For each mental health outcome, we computed post-estimation contrasts to compare odds of the outcome across six pairwise comparisons among patterns of tobacco and cannabis use, using the Bonferroni method to adjust for multiple comparisons. We first used non-use as a reference group for patterns of tobacco and cannabis use. We then assessed the reference group as tobacco-only use or cannabis-only use in order to compare co-use with single-substance use. To further examine the associations between frequency of tobacco and cannabis use and the outcomes, we fit another GEE model for each outcome with daily use of cigarettes, e-cigarettes, other combustible tobacco products, and cannabis. All models accounted for demographic variables, alcohol consumption, and physical activity as these factors were associated with mental health in previous studies [37,38]. Variance inflation factors were less than 2 for all analyses, indicating that multicollinearity was not an issue. All tests were two-tailed with a significance level of α less than 0.05.

We also conducted several sensitivity analyses. First, we ran linear mixed-effects models for continuous mental health outcomes (i.e., GAD-7 and PHQ-8 scores) with the four patterns of tobacco and cannabis use. We then checked dose-response relationships by running linear mixed-effects models with continuous independent variables for tobacco and cannabis use (i.e., number of days using tobacco and cannabis) and continuous mental health outcomes (i.e., GAD-7 and PHQ-8 scores).

## Results

### Participant characteristics

Participants’ baseline characteristics are described in Table 1. The participants had a mean age of 51.0 ± 15.2 (SD) years old. The majority were female (67.9%), non-Hispanic White (81.8%), identified as cisgender (98.6%), and had education attainment of some college or college graduate (51.4%). The mean score of subjective social status was 6.9 ± 1.6 (SD). More than half reported doing exercise more than once a week in the past year. Participants reported having a median of 1 drink of alcohol (IQR = 0–5) in the past week. Regarding use patterns of tobacco and cannabis, 2,617 participants (4.9% of the sample) reported tobacco-only use; 3,702 (6.9%) reported cannabis-only use, 889 (1.6%) reported co-use, and 46,633 (86.6%) reported non-use. In terms of mental health, 6428 (11.9%) and 6794 (12.6%) participants reported experiencing anxiety and depression in at least one monthly survey, respectively. Proportions of self-reported anxiety and depression were highest among those who reported co-use of tobacco and cannabis (26.5% and 28.3%, respectively) and lowest among those who reported non-use (10.6% and 11.2%, respectively).

**Table 1:** Characteristics of the total sample and by patterns of tobacco and cannabis.

Characteristics	Total (N = 53,843)	Tobacco-only use (n = 2,617)	Cannabis-only use (n = 3,702)	Co-use (n = 889)	Non-use (n = 46,633)	p value
**Age**, mean (SD)	51.0 (15.2)	49.1 (14.1)	46.2 (15.1)	43.7 (13.9)	51.7 (15.1)	<0.001
**Age groups**, n (%)						
18–29	4,009 (7.5)	184 (7.0)	521 (14.1)	145 (16.3)	3,159 (6.8)	<0.001
30–39	10,908 (20.3)	596 (22.8)	992 (26.8)	256 (28.7)	9,064 (19.4)	
40–49	10,226 (19.0)	601 (23.0)	687 (18.6)	180 (20.2)	8,758 (18.8)	
50–59	10,934 (20.3)	559 (21.4)	583 (15.8)	157 (17.6)	9,635 (20.7)	
60–69	10,555 (19.6)	433 (16.6)	660 (17.8)	122 (13.7)	9,340 (20.0)	
70+	7,168 (13.3)	242 (9.3)	257 (6.9)	29 (3.3)	6,640 (14.2)	
**Sex assigned at birth**, n (%)						
Male	17,230 (32.1)	1,045 (39.9)	1,325 (35.8)	358 (40.2)	14,502 (31.1)	<0.001
Female	36,531 (67.9)	1,568 (59.9)	2,364 (63.9)	531 (59.6)	32,068 (68.8)	
**Gender identity**, n (%)						
Cisgender	53,082 (98.6)	2,580 (98.6)	3,561 (96.2)	856 (96.1)	46,085 (98.8)	<0.001
Transgender and non-binary gender	659 (1.2)	32 (1.2)	131 (3.5)	33 (3.7)	463 (1.0)	
**Race and Ethnicity**, n (%)						
Non-Hispanic White	44,065 (81.8)	2,094 (80.0)	2,956 (79.9)	694 (77.9)	38,321 (82.2)	<0.001
Non-Hispanic Black	1,245 (2.3)	76 (2.9)	103 (2.7)	27 (3.0)	1,039 (2.2)	
Non-Hispanic Asian	3,110 (5.8)	95 (3.6)	169 (4.6)	36 (4.0)	2,810 (6.0)	
Non-Hispanic Other	1,110 (2.1)	78 (3.0)	98 (2.6)	36 (4.0)	898 (1.9)	
Hispanic	3,877 (7.2)	246 (9.4)	350 (9.5)	89 (10.0)	3,192 (6.8)	
**Education attainment**, n (%)						
High school or less	1,783 (3.3)	229 (8.8)	140 (3.8)	78 (8.8)	1,336 (2.9)	<0.001
Some college or college graduate	27,698 (51.4)	1,676 (64.0)	2,101 (56.7)	590 (66.2)	23,331 (50.0)	
Postgraduate	23,842 (44.3)	679 (26.0)	1,419 (38.3)	206 (23.1)	21,538 (46.2)	
Other/Unknown	510 (1.0)	33 (1.2)	42 (1.1)	16 (1.8)	419 (0.9)	
**Subjective social status ladder,** mean (SD)	6.9 (1.6)	6.2 (1.8)	6.6 (1.7)	5.9 (1.8)	7.0 (1.6)	<0.001
**Past-year exercise frequency,** n (%)						
Never or rarely	4,066 (7.6)	381 (14.6)	253 (6.8)	102 (11.4)	3,330 (7.1)	<0.001
Less than once a month	4,775 (8.8)	334 (12.8)	323 (8.7)	105 (11.8)	4,013 (8.6)	
Less than once a week	6,608 (12.3)	385 (14.7)	468 (12.6)	153 (17.2)	5,602 (12.0)	
About once a week	6,544 (12.1)	398 (15.2)	468 (12.6)	138 (15.5)	5,540 (11.9)	
Less than 4 times a week	15,330 (28.5)	602 (23.0)	1,152 (31.1)	218 (24.5)	13,358 (28.6)	
4 or more times a week	16,263 (30.2)	502 (19.2)	1,026 (27.7)	173 (19.4)	14,562 (31.2)	
Other	239 (0.4)	13 (0.5)	11 (0.3)	2 (0.2)	213 (0.5)	
**Number of alcohol drinks in the past week,** median (IQR)	1 (0–5)	2 (0–7)	3 (0–7)	3 (0–10)	1 (0–5)	<0.001
**Number of monthly surveys completed**						
Anxiety surveys	354,721	13,960	22,031	4,444	314,286	
Depression surveys	353,444	13,896	21,953	4,431	313,164	
**Reported mental health disorders in at least one monthly survey,** n (%)						
Anxiety	6,428 (11.9)	503 (19.2)	752 (20.3)	236 (26.5)	4,937 (10.6)	<0.001
Depression	6,794 (12.6)	571 (21.8)	769 (20.8)	252 (28.3)	5,202 (11.2)	<0.001

Note: Proportion by column; Missing values (n, %): Age (43, 0.08%); Sex (82, 0.15%); Gender identity (102, 0.19%), Race/ethnicity (436, 0.81%), Education (10, 0.02%); Social ladder (7, 0.01%); Exercise (18, 0.03%); Alcohol use (8,969; 16.7%).

#### Association between patterns of tobacco and cannabis use and mental health outcomes

Results from the GEE models are presented in Table 2. Compared to non-use, the adjusted odds of mental health disorders were highest for co-use (*Anxiety*: *OR 1*.*89*, *95% CI 1*.*64–2*.*18; Depression*: *OR 1*.*77*, *95% CI 1*.*46–2*.*16*), followed by cannabis-only use (*Anxiety*: *OR 1*.*65*, *95% CI 1*.*52–1*.*78; Depression*: *OR 1*.*67*, *95% CI 1*.*50–1*.*86)*, and tobacco-only use *(Anxiety*: *OR 1*.*40*, *95% CI 1*.*28–1*.*54; Depression*: *OR 1*.*45*, *95% CI 1*.*28–1*.*64)*.

**Table 2:** Association between patterns of tobacco and cannabis use and anxiety and depression.

Independent variables	Anxiety (GAD7≥10) OR (95%CI)	Depression (PHQ8≥10) OR (95%CI)
**Comparisons between patterns of tobacco and cannabis use**		
**Comparison to Non-use**		
Co-use vs. Non-use	1.89 (1.64, 2.18)***	1.77 (1.46, 2.16)***
Cannabis-only vs. Non-use	1.65 (1.52, 1.78)***	1.67 (1.50, 1.86)***
Tobacco-only vs. Non-use	1.40 (1.28, 1.54)***	1.45 (1.28, 1.64)***
**Comparison to Tobacco-only use**		
Co-use vs. Tobacco-only use	1.35 (1.08, 1.69)**	1.22 (0.98, 1.53)
Cannabis-only vs. Tobacco-only use	1.17 (1.00, 1.37)*	1.15 (0.98, 1.35)
**Comparison to Cannabis-only use**		
Co-use vs. Cannabis-only use	1.15 (0.93, 1.42)	1.06 (0.86, 1.32)
**Covariates**		
**Age groups**		
18–29	1.00 [Reference]	1.00 [Reference]
30–39	0.80 (0.73, 0.86)***	0.81 (0.75, 0.88)***
40–49	0.66 (0.61, 0.72)***	0.69 (0.63, 0.75)***
50–59	0.43 (0.39, 0.47)***	0.49 (0.45, 0.54)***
60–69	0.28 (0.25, 0.30)***	0.32 (0.29, 0.35)***
70+	0.15 (0.13, 0.17)***	0.19 (0.16, 0.21)***
**Female vs. Male**	1.49 (1.41, 1.58)***	1.34 (1.26, 1.41)***
**Transgender and non-binary gender vs. Cisgender**	2.10 (1.81, 2.43)***	2.44 (2.10, 2.85)***
**Race and Ethnicity**		
** **Non-Hispanic White	1.00 [Reference]	1.00 [Reference]
Non-Hispanic Black	0.75 (0.64, 0.88)***	0.70 (0.60, 0.82)***
Non-Hispanic Asian	0.78 (0.70, 0.87)***	0.75 (0.67, 0.84)***
Non-Hispanic Other	1.15 (0.99, 1.33)	1.22 (1.06, 1.42)**
Hispanic	1.05 (0.97, 1.14)	1.01 (0.93, 1.09)
**Education attainment**		
Highschool or less	1.00 [Reference]	1.00 [Reference]
Some college or College graduate	0.93 (0.83, 1.04)	0.95 (0.85, 1.06)
Postgraduate	1.00 (0.89, 1.13)	0.90 (0.80, 1.02)
**Subjective social status ladder (from 1 to 10)**	0.82 (0.81, 0.83)***	0.78 (0.77, 0.79)***
**Number of alcohol drinks**	1.01 (1.00, 1.01)*	1.00 (1.00, 1.00)
**Frequency of physical activity**	0.90 (0.89, 0.91)***	0.82 (0.81, 0.84)***
**Year of data collection**		
2020	1.00 [Reference]	1.00 [Reference]
2021	0.65 (0.63, 0.67)***	0.80 (0.78, 0.82)***
2022	0.64 (0.62, 0.66)***	0.83 (0.80, 0.86)***

Note: Significance level:

***: p<0.001;

**: p<0.01;

*: p<0.05.

OR: Odd Ratio. CI: Confidence Interval. The models included all the independent variables simultaneously. N for the anxiety models: N = 269,869 observations (43,972 participants); N for the depression models: N = 268,926 observations (43,877 participants). Subjective social status, alcohol drinks, and frequency of physical activity were continuous variables, with higher values corresponding to higher subjective social status, greater alcohol consumption, or having greater frequency of physical activity.

Compared to tobacco-only use, co-use was associated with higher adjusted odds for anxiety *(OR 1*.*35*, *95% CI 1*.*08–1*.*69)*, but not for depression *(OR 1*.*22*, *95% CI 0*.*98–1*.*53)*. Likewise, cannabis-only use was associated with higher adjusted odds for anxiety (*OR 1*.*17*, *95% CI 1*.*00–1*.*37)*, but not for depression (*OR 1*.*15*, *95% CI 0*.*98–1*.*35)* compared to tobacco-only use. However, there were no significant differences between co-use and cannabis-only use *(Anxiety*: *OR 1*.*15*, *95% CI 0*.*93–1*.*42; Depression*: *OR 1*.*06*, *95% CI 0*.*86–1*.*32)*.

Several covariates were significantly associated with mental health outcomes. Young adults aged 18–29 years (vs. older age), females (vs. males), and transgender and non-binary gender participants (vs. cisgender peers) had higher odds of reporting anxiety and depression. Conversely, covariates associated with lower odds of having mental health disorders were being non-Hispanic Black, non-Hispanic Asian, or non-Hispanic Other (vs. non-Hispanic White), perceiving higher subjective social status, or having greater frequency of physical activity. Reporting greater alcohol consumption was associated with slightly higher odds of anxiety, but not depression. Across the study years, the odds of reporting anxiety and depression were lower in 2021 and 2020 compared to those in 2020.

#### Association between daily use of tobacco and cannabis and mental health outcomes

Table 3 presents the associations between daily use of tobacco and cannabis products and anxiety and depression. Compared to non-daily use, daily use of cigarettes *(Anxiety*: *OR 1*.*42*, *95% CI 1*.*24–1*.*61; Depression*: *OR 1*.*41*, *95% CI 1*.*24–1*.*60)* and e-cigarettes *(Anxiety*: *OR 1*.*47*, *95% CI 1*.*25–1*.*74; Depression*: *OR 1*.*59*, *95% CI 1*.*34–1*.*87)* were associated with higher odds of both anxiety and depression. However, the associations between daily use of other combustible tobacco products and the mental health outcomes were not statistically significant. Daily use of cannabis (vs. non-daily use) was associated with higher odds of anxiety (*OR 1*.*82*, *95% CI 1*.*58–2*.*10)* and depression (*OR 1*.*68*, *95% CI 1*.*46–1*.*95)*.

**Table 3:** Association between daily use of tobacco and cannabis products and anxiety and depression.

Independent variables	Anxiety (GAD7≥10) OR (95%CI)	Depression (PHQ8≥10) OR (95%CI)
**Tobacco use**		
Daily use of cigarettes (Yes vs. No)	1.42 (1.24, 1.61)***	1.41 (1.24, 1.60)***
Daily use of e-cigarettes (Yes vs. No)	1.47 (1.25, 1.74)***	1.59 (1.34, 1.87)***
Daily use of other combustible tobacco products (Yes vs. No)	1.00 (0.58, 1.74)	0.83 (0.48, 1.46)
**Daily use of cannabis** (Yes vs. No)	1.82 (1.58, 2.10)***	1.68 (1.46, 1.95)***
**Age groups**		
18–29	1.00 [Reference]	1.00 [Reference]
30–39	0.78 (0.72, 0.84)***	0.79 (0.73, 0.86)***
40–49	0.64 (0.59, 0.70)***	0.67 (0.61, 0.73)***
50–59	0.41 (0.38, 0.45)***	0.48 (0.43, 0.52)***
60–69	0.26 (0.24, 0.29)***	0.31 (0.28, 0.34)***
70+	0.14 (0.12, 0.16)***	0.18 (0.15, 0.20)***
**Female vs. Male**	1.48 (1.40, 1.56)***	1.32 (1.25, 1.39)***
**Transgender and non-binary gender vs. Cisgender**	2.20 (1.90, 2.55)***	2.52 (2.16, 2.93)***
**Race and Ethnicity**		
** **Non-Hispanic White	1.00 [Reference]	1.00 [Reference]
Non-Hispanic Black	0.78 (0.67, 0.91)**	0.73 (0.63, 0.85)***
Non-Hispanic Asian	0.78 (0.70, 0.87)***	0.75 (0.67, 0.84)***
Non-Hispanic Other	1.17 (1.01, 1.35)*	1.26 (1.09, 1.45)**
Hispanic	1.07 (0.99, 1.16)	1.02 (0.94, 1.11)
**Education attainment**		
Highschool or less	1.00 [Reference]	1.00 [Reference]
Some college or College graduate	0.93 (0.82, 1.04)	0.94 (0.84, 1.06)
Postgraduate	1.00 (0.89, 1.13)	0.89 (0.79, 1.01)
**Subjective social status ladder (from 1 to 10)**	0.82 (0.81, 0.83)***	0.78 (0.77, 0.79)***
**Number of alcohol drinks**	1.01 (1.00, 1.01)***	1.00 (1.00, 1.01)
**Frequency of physical activity**	0.90 (0.89, 0.91)***	0.83 (0.81, 0.84)***
**Year of data collection**		
2020	1.00 [Reference]	1.00 [Reference]
2021	0.65 (0.63, 0.67)***	0.80 (0.78, 0.82)***
2022	0.64 (0.62, 0.67)***	0.83 (0.80, 0.86)***

Note: Significance level:

***: p<0.001;

**: p<0.01;

*: p<0.05.

OR: Odd Ratio. CI: Confidence Interval. The models included all the independent variables simultaneously. N for the anxiety models: N = 269,869 observations (43,972 participants); N for the depression models: N = 268,926 observations (43,877 participants). Subjective social status, alcohol drinks, and frequency of physical activity were continuous variables, with higher values corresponding to higher subjective social status, greater alcohol consumption, or having greater frequency of physical activity.

Results of the sensitivity analyses were very similar to those of the primary analyses. Compared to non-use, co-use was associated with the highest scores of GAD-7 and PHQ-8, followed by cannabis-only use, and tobacco-only use (S1 Table). Co-use and cannabis-only were associated with higher scores for GAD-7 and PHQ-8 than tobacco-only use. However, co-use was associated with higher scores of GAD-7 and PHQ-8 than cannabis-only use. Regarding dose-response relationships, greater frequency of using cigarettes, e-cigarettes, and cannabis were associated with higher scores of GAD-7 and PHQ-8 (S2 Table).

## Discussion

### Principal results

This study examined the extent to which different patterns of tobacco and cannabis use are associated with mental health disorders among US adults enrolled in a digital cohort study. As hypothesized, we found that use of tobacco and/or cannabis was positively associated with self-reported anxiety and depression. Notably, co-use was associated with higher odds of anxiety when compared to tobacco-only use, but not different from cannabis-only use. We also found that daily use of cigarettes, e-cigarettes, and cannabis were associated with the increased odds of reporting anxiety and depression. To our knowledge, this is the first study among the US adult population to evaluate the association of mental health with four mutually exclusive patterns of tobacco and cannabis use, with a focus on co-use of both substances.

### Comparison with prior work

Consistent with prior studies [22,30], our study found that using tobacco alone and using cannabis alone were associated with higher odds of mental health disorders. Our study further extends the literature by indicating a graded effect size, ranging from lowest for tobacco-only, cannabis-only, to highest for co-use when comparing to non-use. This finding suggests both independent effects of tobacco and cannabis and joint effects of both substances on mental health. However, we could not determine causation of these associations due to the lack of data on initiation of tobacco and cannabis use as well as temporal onset of anxiety and depression. Current literature on this area is mixed, and accumulating evidence supports a bidirectional relationship between use of tobacco and cannabis and mental health [14]. Three mechanisms are proposed to explain the relationship between substance use and mental health, which could also be employed to explain our observed associations between use of tobacco and/or cannabis and anxiety and depression. These mechanisms are: (1) the self-medication or coping mechanism (using tobacco/cannabis to self-regulate and mitigate anxiety and depressive symptoms); (2) the substance-induced mechanism (use of tobacco and cannabis leads to a toxic effect and neurological changes that prompt or exacerbate anxiety and depressive symptoms, particularly during periods of acute or prolonged withdrawal); and (3) the shared-vulnerability mechanism (common genetic, behavioral, and environmental factors that predispose individuals to both substance use and mental health problems) [14]. Future work utilizing longitudinal research designs is needed to elucidate whether causal pathways differ for patterns of tobacco and cannabis use in the relationship with mental health disorders, ultimately informing prevention and treatment efforts.

Compared to single substance use, the odds of anxiety for co-use were higher than those for tobacco-only use, but were not significantly different from cannabis-only use. While these findings are suggestive of an additive effect of cannabis use on mental health among people who use tobacco, an additive effect of tobacco use among those who use cannabis use was not certain. This may be related to the large proportion of cannabis-only use in our sample. Indeed, 80.6% of our participants with past 30-day cannabis use reported using cannabis-only, while this proportion was only 8.4% in a population-based sample [39]. It should be noted that in our sensitivity analysis, co-use was significantly associated with higher scores of GAD-7 and PHQ-8 than cannabis-only use. Future studies are needed to confirm the robustness of our findings in other samples.

In addition, we found that lower frequency of physical activity was associated with higher odds of anxiety and depression. A review indicates that both tobacco use and low physical activity are lifestyle risk factors for mental health [40]. It should be noted that physical activity could be also a mediator in the relationship between substance use and mental health since people who use tobacco or cannabis may engage in less physical activity, which in turn results in poorer mental health. Given this study focused on the association between co-use and mental health, we adjusted for physical activity as a covariate in the multivariate models, and did not examine a potential meditating effect of physical activity. Future research should explore potential pathways through which multiple lifestyle factors may influence mental health outcomes.

As this study is conducted from early (2020) to later periods (2022) in the COVID-19 pandemic, we also found that the odds of reporting anxiety and depression were lower in later years than in 2020. This finding could be partly explained by unprecedented stress, economic hardship, lockdowns, and social isolation early in the pandemic [41,42].

### Study implications

Our study has implications for clinical practice and public health efforts. The observed associations between use of tobacco and/or cannabis and anxiety and depression call for more attention to the comorbidity of substance use and mental health. This comorbidity is particularly concerning among those who use both tobacco and cannabis. Increased vulnerability to poor mental health in the co-use group may impede successful cessation from tobacco and cannabis [27]. Furthermore, co-use is common in low socioeconomic and sexual minority subpopulations, and thus, could exacerbate health disparities related to tobacco and cannabis use [43,44]. In addition to use of tobacco and/or cannabis, we also found that greater alcohol consumption was associated with higher odds of anxiety. Alcohol is frequently used with tobacco and cannabis and hazardous drinking is more prevalent among people with co-use compared to those with single substance use [45]. Polysubstance use may compromise the success of cessation from tobacco and cannabis [46]. As such, our study highlights a need for providing mental health support and addressing polysubstance use issues among people with co-use of tobacco and cannabis.

As tobacco and cannabis use are common among people with mental health issues, screening for use of tobacco and cannabis should be a priority in mental health treatment settings. In California, universal screening for tobacco use in substance use treatment settings was signed into law in 2021 [47]. In regards to treatment, our study suggests that coordinating tobacco and cannabis cessation with mental health treatment may be beneficial. Specifically, providing alternative coping strategies to reduce depressive and anxiety symptoms may be beneficial to people with use of tobacco and/or cannabis. A systematic review found that adding psychosocial mood management to usual smoking cessation treatment for people with current and historical depression increased smoking abstinence [48]. Similarly, counseling to support tobacco and cannabis cessation may improve mental health [49,50]. Indeed, another systematic review and meta-analysis showed that smoking cessation is associated with reduced anxiety, depression, and stress and improved mood and quality of life [51]. As we found that daily use of tobacco and cannabis is associated with higher odds of anxiety and depression, cessation services for people with mental health disorders should be tailored to address heavy use and dependence of nicotine and cannabis.

Furthermore, despite insufficient evidence regarding therapeutic benefits of cannabis, nearly half of US adults view cannabis as self-medication for treating depression and anxiety symptoms [52]. Similarly, many people, especially young adults, misinterpret nicotine as helping to relieve stress, anxiety, and depression [53]. To correct these misperceptions, public health campaigns should highlight potential negative effects of tobacco and cannabis use on mental health.

### Limitations

This study has several limitations. As aforementioned, the causal relationships between patterns of tobacco and cannabis use and mental health disorders cannot be elucidated given the study design. Since use of tobacco and cannabis was measured only once at baseline, we could not examine potential effects of changes in substance use on mental health. Longitudinal studies using repeated measures of both substance use and mental health over time are needed to uncover causal and dynamic relationships. Our digital cohort recruited via a convenience sampling method which, although allowing for a large sample size and facilitating data collection during the COVID-19 pandemic, may limit the findings’ generalizability. Since our sample is not a nationally representative population-based sample, cautions should be taken in interpreting the findings to other samples. Of note, we only asked about use of combustible or vaporized cannabis products, and thus, findings may not generalize to other modes of consumption. Self-reported data and non-respondents may be subject to bias. Data on nicotine and cannabis dependence and the purpose of cannabis use (e.g., recreational or medicinal cannabis use) were not collected; thus, we could not examine potential impacts of these factors on mental health.

## Conclusions

This study provided recent data on positive associations of the mutually exclusive patterns of tobacco and cannabis use with mental health, with a focus on co-use of both substances among US adults. Co-use of tobacco and cannabis and use of cannabis-only were associated with higher odds of anxiety and depression compared to non-use and tobacco-only use. Tobacco-only use was associated with higher odds of anxiety and depression compared to non-use. The findings suggest that coordinating tobacco and cannabis cessation with mental health treatment may be beneficial to address these comorbidities. Specifically, providing mental health support and addressing polysubstance use (e.g., tobacco, cannabis, alcohol) among individuals with co-use are needed to facilitate successful cessation from tobacco and cannabis.

## Supporting information

S1 Table Comparisons of GAD-7 and PHQ-8 scores between patterns of tobacco and cannabis use. (DOCX)

S2 Table Dose-response relationship between frequency of use of tobacco and cannabis products with GAD-7 and PHQ-8 scores. (DOCX)

S1 Dataset (CSV)
