# Supplementary material for: Associations between tobacco and cannabis use and anxiety and depression among adults in the United States: Findings from the COVID-19 citizen science study
Source: PLoS One. 2023 Sep 13;18(9):e0289058. doi: 10.1371/journal.pone.0289058 (PMC10499225; doi:10.1371/journal.pone.0289058)
Supplement: S1 Table — (DOCX) [file pone.0289058.s001.docx]

**S1 Table: Comparisons of GAD-7 and PHQ-8 scores between patterns of tobacco and cannabis use**

| **Comparisons** | **GAD-7 Score**  β (95%CI) | **PHQ-8 Score**  β (95%CI) |
| --- | --- | --- |
| **Comparison to Non-use** |  |  |
| Co-use vs. Non-use | 1.67 (1.37, 1.96)*** | 1.70 (1.39, 2.00)*** |
| Cannabis-only vs. Non-use | 1.17 (1.02, 1.32)*** | 1.30 (1.15, 1.45)*** |
| Tobacco-only vs. Non-use | 0.72 (0.54, 0.89)*** | 0.91 (0.73, 1.08)*** |
| **Comparison to Tobacco-only use** |  |  |
| Co-use vs. Tobacco-only use | 0.95 (0.62, 1.28)*** | 0.79 (0.45, 1.13)*** |
| Cannabis-only vs. Tobacco-only use | 0.45 (0.23, 0.67)*** | 0.39 (0.17, 0.62)** |
| **Comparison to Cannabis-only use** |  |  |
| Co-use vs. Cannabis-only use | 0.49 (0.17, 0.82)** | 0.40 (0.06, 0.73)* |

Note: Significance level: ***: p<0.001; **: p<0.01; *: p<0.05. CI: Confidence Interval.

All mixed-effect models were adjusted for the same covariates as those in Table 2.

GAD-7 score ranged from 0 to 21. PHQ-8 score ranged from 0 to 24
