# Supplementary material for: Associations between tobacco and cannabis use and anxiety and depression among adults in the United States: Findings from the COVID-19 citizen science study
Source: PLoS One. 2023 Sep 13;18(9):e0289058. doi: 10.1371/journal.pone.0289058 (PMC10499225; doi:10.1371/journal.pone.0289058)
Supplement: S2 Table — (DOCX) [file pone.0289058.s002.docx]

**S2 Table: Dose-response relationship between frequency of use of tobacco and cannabis products**

**with GAD-7 and PHQ-8 scores**

| **Independent variables** | **GAD-7 Score**  β (95%CI) | **PHQ-8 Score**  β (95%CI) |
| --- | --- | --- |
| **Frequency of tobacco use in the past 30 days** |  |  |
| Number of days using cigarettes | 0.03 (0.02, 0.04)*** | 0.03 (0.02, 0.04)*** |
| Number of days using e-cigarettes | 0.04 (0.03, 0.05)*** | 0.05 (0.04, 0.06)*** |
| Number of days using other combustible tobacco products | -0.01 (-0.04, 0.01) | -0.01 (-0.03, 0.02) |
| **Frequency of cannabis use in the past 30 days** |  |  |
| Number of days using combustible and vaporized cannabis products | 0.06 (0.05, 0.07)*** | 0.07 (0.06, 0.08)*** |
| **Age groups** |  |  |
| 18-29 | 0.00 [Reference] | 0.00 [Reference] |
| 30-39 | -0.46 (-0.62, -0.29)*** | -0.60 (-0.77, -0.43)*** |
| 40-49 | -1.13 (-1.29, -0.96)*** | -1.06 (-1.23, -0.89)*** |
| 50-59 | -2.23 (-2.40, -2.07)*** | -1.88 (-2.05, -1.71)*** |
| 60-69 | -3.10 (-3.27, -2.94)*** | -2.72 (-2.89, -2.55)*** |
| 70+ | -3.71 (-3.89, -3.54)*** | -3.24 (-3.42, -3.06)*** |
| **Female vs. Male** | 0.98 (0.90, 1.06)*** | 0.72 (0.64, 0.80)*** |
| **Non-binary gender vs. Binary gender** | 2.22 (1.87, 2.56)*** | 2.66 (2.30, 3.01)*** |
| **Race and Ethnicity** |  |  |
| Non-Hispanic White | 0.00 [Reference] | 0.00 [Reference] |
| Non-Hispanic Black | -0.86 (-1.10, -0.62)*** | -0.95 (-1.19, -0.71)*** |
| Non-Hispanic Asian | -0.73 (-0.90, -0.57)*** | -0.74 (-0.91, -0.57)*** |
| Non-Hispanic Other | 0.14 (-1.22, 0.40) | 0.43 (0.16, 0.70)** |
| Hispanic | 0.03 (-0.12, 0.17) | 0.00 (-0.15, 0.15) |
| **Education attainment** |  |  |
| Highschool or less | 0.00 [Reference] | 0.00 [Reference] |
| Some college or College graduate | 0.06 (-0.15, 0.27) | 0.02 (-0.20, 0.23) |
| Postgraduate | 0.29 (0.08, 0.51)** | 0.01 (-0.21, 0.22) |
| **Subjective social status ladder** | -0.41 (-0.43, -0.38)*** | -0.51 (-0.54, -0.49)*** |
| **Number of alcohol drinks** | 0.02 (0.01, 0.02)*** | 0.01 (0.00, 0.02)* |
| **Frequency of physical activity** | -0.19 (-0.21, -0.16)*** | -0.41 (-0.43, -0.38)*** |

*Note:* Significance level: ***: p<0.001; **: p<0.01; *: p<0.05. The mixed-effects models included all variables simultaneously. N for the anxiety models: N=269,869 observations (43,972 participants); N for the depression models: N=268,926 observations (43,877 participants). GAD-7 score ranged from 0 to 21. PHQ-8 score ranged from 0 to 24. Frequency of each tobacco product was asked separately, while frequency of cannabis use was asked as number of days using any vaporized or combustible cannabis.
